# Investigating the referral of patients with non-urgent conditions to a regional Australian emergency department: a study protocol

**DOI:** 10.1186/s12913-018-3411-4

**Published:** 2018-08-20

**Authors:** Maria Unwin, Elaine Crisp, Scott Rigby, Leigh Kinsman

**Affiliations:** 1 College of Health and Medicine, University of Tasmania c/- Level 2, Northern Integrated Care Services, 41 Frankland St, Launceston, TAS 7250 Australia; 2 Emergency Department, Launceston General Hospital, Tasmanian Health Service, 274–280 Charles St, Launceston, Tasmania Australia; 3 College of Health and Medicine, University of Tasmania, 1 Newham Close, Newnham, Tasmania 7248 Australia; 4 Tasmanian Health Service c/-Level 2, Northern Integrated Care Services, 41 Frankland St, Launceston, Tasmania 7250 Australia

**Keywords:** Emergency department, Non-urgent presentations, Referral, Primary care, General practitioner

## Abstract

**Background:**

Australia’s only island state, Tasmania, experiences one of the nation’s highest incidences of non-urgent emergency department (ED) presentations in a healthcare system regularly faced with service demands that exceed resource availability. Service-demand mismatches are acknowledged to contribute to ED crowding which in turn, has been documented to have a correlation with poorer patient outcomes. Crowding within EDs is complex, non-urgent presentations alone are not the primary cause, but have been reported to be a contributing factor. In 2015–16 Tasmania recorded over 153,000 ED attendances, 55% of these fell into the two least urgent triage categories. Recent research in the State’s North established that 29% of non-urgent presentations were referred, formally or informally, from primary healthcare providers and that, for many patients (39%), the ED was not their first choice of service provider. This study aims to identify the service needs of patients referred to a regional Australian ED and subsequently triaged as non-urgent.

**Method:**

In order to achieve this aim, three objectives have been identified. The first two objectives use an explanatory sequential mixed-method approach while the third objective will incorporate an implementation science approach. These three objectives are: first, a retrospective analysis of seven years of routinely collected hospital data to identify trends in referral of patients with non-urgent conditions; second, focus group interviews with patients and primary care providers to further understand perceived need and service requirements of those referred to the ED, and third, translation of findings into local health service recommendations.

**Discussion:**

Identification of the needs of patients referred to the ED with non-urgent conditions will inform future service planning aiming to facilitate access to the right service at the right time and in the right place.

## Background

Worldwide interest in the demand for emergency department (ED) services is evidenced by a growing body of work demonstrating links between ED crowding and patient outcomes. Crowding occurs when the demand for services exceeds resource and space availability, and has been linked to negative consequences for both patients and the healthcare system. In 2000, Derlet and Richards [1] identified a number of concerns held by ED physicians across the United States which included: increased risk to public safety; increased time to analgesia; extended waiting time; patient dissatisfaction; decreased physician satisfaction; increased violence; miscommunication; and negative impact on teaching. Since then, these themes have remained constant; with increased hospital length of stay, morbidity and mortality also shown to be associated with ED crowding [2–8]. A Canadian team in 2014 [3] conducted a retrospective analysis of over 600,000 ED presentations to 42 hospitals, and they reported significant risks to patient safety occurring during periods of crowding. To date, there is considerable evidence indicating links between ED crowding and poorer outcomes for patients, but there appears to be less knowledge around the causes driving patients to attend EDs. These drivers have been referred to as ED input factors [9]. Recent studies have demonstrated a link between ED crowding and the presence of patients with non-urgent conditions in the ED and limited access to primary care services [10–14].

In 2017, Crawford and colleagues [15] published a systematic review and discussed the increase in non-urgent presentations (input factor) and the growing demand placed on EDs, worldwide, by potentially avoidable presentations. Much debate exists over whether these presentations add a significant burden to the workload and resource demands of crowded EDs, with some arguing they do not add a significant burden [16–18]. In Australia, attendances by patients triaged into the two least urgent categories have continued to exceed 50% nationwide [19–22], it is timely to consider the healthcare needs of this patient group and whether alternative models might lead to improved access to timely care and ultimately, to better patient outcomes. Research conducted in Switzerland and Australia [23–25] have reported a younger demographic amongst patients with non-urgent conditions with the most common presenting complaint among these patients being musculoskeletal. Furthermore, two studies [13, 26] report considerable discrepancies between patients’ reasons for attending versus clinicians’ perception of the reasons for ED usage by patients presenting with non-urgent conditions. Durand and colleagues [13] concluded that thorough investigation of the healthcare demand is required before strategies are planned and implemented.

Compounding the issue is the lack of a universal definition of ‘non-urgent ED presentations’; within the Australian context these are most frequently referred to as those presentations allocated the least urgent triage categories of 4 or 5 [24, 26–28] on arrival. Furthermore, a literature review by Forero and colleagues [29] reviewing the ATS discussed the complexities of classifying patients triaged as ATS 4 and 5 as ‘primary-care suitable’, ‘general-practitioner type’ or ‘inappropriate’; however, for the purposes of this study, the research team include all patients triaged as ATS 4 or 5. The authors acknowledge that this patient group, considered to have non-urgent conditions, will include patients presenting with both low-urgency needs who are unsuitable for primary care and those who are potentially suitable for primary care. Recent Tasmanian research has demonstrated that if primary care services were available at the time of need in regional Northern Tasmania this could result in up to 8000 less ED presentations annually [24].

An Italian research team conducted a retrospective cohort study and identified excessive referrals of patients with non-urgent conditions as a contributor to ED crowding [12]. These authors identified that few studies have considered referrals to ED and how such referrals may contribute to crowding. The question of where to best manage the needs of this patient group has not been clearly answered. This is a concern for healthcare providers who face growing demands for services, and for patients who may experience poorer health outcomes in crowded EDs [2, 3, 8].

In Australia, between July 2011 and June 2016 the percentage of ED patients triaged as ATS 4 and 5 has continued to exceed 51% of total ED presentations. From June 2015 to July 2016 these non-urgent presentations totalled over 3.8 million nationwide [22]. Tasmania has one the highest incidences of non-urgent ED presentations at 55.3%. In Australia, residents are free to choose between their General Practitioner (GP) and ED services for management of their acute, non-urgent conditions. GP services provide a limited number of same-day appointments, and once these are fully allocated patients must consider alternatives, of which ED is perceived as a convenient option [24]. Additionally, there are a small number of privately run GP services that provide after-hours services. Research from the UK demonstrated that commencement of a co-located after-hours clinic reduced ED presentations [10], yet a systematic review by Crawford and colleagues concluded that evidence on the effect of GP walk-in centres was infrequent and further research is required to determine the proficiency of services as alternatives to EDs [15].

This project has arisen out of research conducted in 2015 at a regional Tasmanian ED in which the researchers [24] identified that 39% of patients with non-urgent conditions had attempted to access alternative healthcare services before arriving at the ED. This surveyed patient group also indicated that 31% would have preferred to be managed by their GP. These findings demonstrated that the ED is not necessarily the first point of contact, nor in fact, the first preference of this patient group. Furthermore, 29% of patients with non-urgent conditions were referred to the ED by a healthcare provider. The term ‘referral’ used in this instance, includes both formal and informal referrals. The findings of this project will provide greater understanding of local issues and service needs.

Variation in health-seeking behaviour across Tasmanian regions was identified by Morley and colleagues who were able to demonstrate that despite its small geographical and population size, each of Tasmania’s three regions (South, North and Northwest) contribute a unique profile to the State’s ED attendances [30]. They concluded that future research needs to consider factors driving the various trends and implement services specific to regional demands. This project will provide a local, contextually relevant picture of the issues driving the demand for non-urgent ED presentations in Northern Tasmania.

This study will aim to identify the service requirements of patients with non-urgent conditions referred, formally or informally, to a regional Australian ED. The objectives to address this aim are: first, to identify trends in primary care referral of non-urgent patients to a regional Tasmanian ED over the previous 7 years; second, to identify the perceived need and service requirements of patients referred from primary care to ED; and third, to translate findings into local health service recommendations.

## Methods

### Overall design

**Fig. 1:**
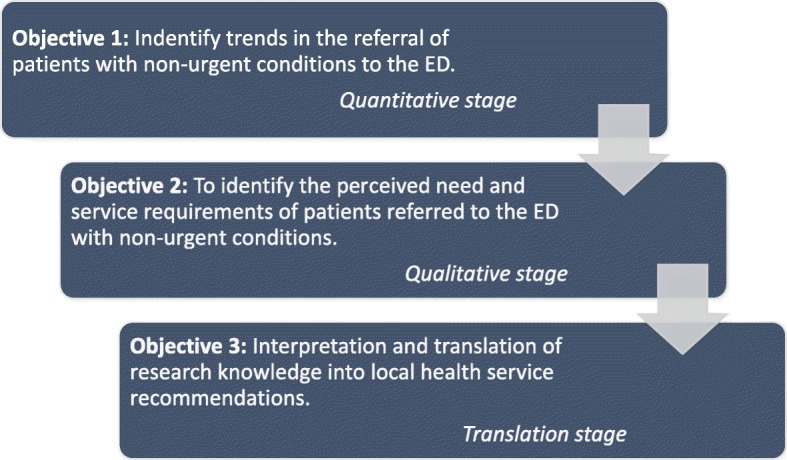
Project flow-diagram for ‘Primary care to emergency department (ED): right service, right time, right place’

In order to achieve the aim of identifying service requirements of patients who have been referred, formally or informally, with non-urgent conditions to a regional Tasmanian ED, this project will implement an explanatory sequential mixed-method approach. The primary objective will be to identify trends in the referral of patients with non-urgent conditions to the ED. The second objective will be to identify the perceived need and service requirements of patients referred to the ED with non-urgent conditions, while the third objective will facilitate translation of these findings into health service recommendations. Figure 1 (below) provides a summary of the research plan and is based on Creswell’s design for sequential explanatory mixed methods [31] with the addition of a third objective to disseminate and translate research findings.

### Objective 1: Identification of trends in the referral of patients with non-urgent conditions

The focus of the first objective will be to identify trends in the referral of patients with non-urgent conditions, including changes over time, in order to establish a profile of who, when and why patients have accessed ED services with non-urgent conditions. This will involve the analysis of routinely collected ED attendance records for patients presenting and triaged as ATS 4 or 5 during a seven-year period, from July 2009 to June 2016 at a regional Tasmanian hospital. This data is routinely collected by the Tasmanian Health Service (THS) and stored on a data platform by the Department of Health and Human Service, Tasmania.

The study population for this objective will include all ATS 4 and 5 patients presenting to the ED from July 2009 to June 2016. Data collected will include: date, day of week and time of presentation; age and gender; mode of arrival; triage category on arrival; residential suburb; time to first seen by ED physician or nurse practitioner; total ED length of stay; referral sources into ED and on discharge, and discharge diagnosis and destination. Presentations will be excluded if: their usual place of residence is outside of THS-North’s catchment area.

Once obtained, the data will be entered into a statistical software package (SPSS, V22) [32] and analysed for themes, trends and relationships. An interrupted time series (ITS) analysis will be undertaken to determine whether factors such as the number of available general practitioners within the local area or the opening of an additional after hours, walk-in service has affected the number of patients referred with non-urgent conditions or has influenced the overall number of ED presentations. ITS allows researchers to control for trends when comparing data pre and post an intervention and is known to provide robust quasi-experimental research design [33].

### Objective 2: Identification of perceived needs and service requirements of patients referred to the ED with non-urgent conditions

The second stage of this project will involve focus groups with patients referred to the ED and with primary care providers who have referred patients to the ED. Themes, trends and relationships identified during the first objective will be summarised and presented to participants to facilitate further exploration of the local context and to understand the phenomenon of patients with non-urgent conditions being referred to the ED. All participants will be asked to provide signed consent prior to participating in focus groups.

Focus groups are advantageous in healthcare research, allowing researchers to include representation from various community groups and enabling researchers to investigate participants’ knowledge and experience of situations while engaging in conversations that facilitate exploration of an issue [34]. Based on the nature of this study, the research team plan to conduct homogenous focus groups with a total of eight to 12 patient participants, with a subsequent homogenous GP focus group. The first group will be conducted with participants who have been referred to the ED with non-urgent conditions whilst the second will be with GPs and primary care providers who have referred patients with non-urgent conditions to the ED. Gerrish and Lacey [35] discuss homogenous versus heterogeneous groups and state that homogenous groups can assist facilitation of free discussion; they go on to recommend a group size of five to 12 to facilitate engaged group dialog.

Patient participants will be given an opportunity to discuss their decision-making process and episode of care from the community to the ED. Eligible patients will be provided with brochures by ED clinical staff and will have the opportunity to opt into focus group participation. The intent will be to recruit a stratified representative sample. Based on the profile of non-urgent attendees from our research [24] conducted in 2015, the proposed patient focus group will aim to consist of: two parent participants (whose young children attended the ED as patients); two participants under 25 years of age; three participants between 25 to 64 years of age, and one participant over 65 years of age. Consideration will also be given to focus group participants’ presenting condition (in-line with the profile of non-urgent attendees from previous research) aiming to include a combination of presentations, such as musculoskeletal, general conditions such as headache, cold and flu-like symptoms, and gastrointestinal symptoms [24].

A purposive sample of GPs referring patients to the ED will be invited to attend the second clinician focus group. This group will consist of six to eight clinicians from a range of medical practices within the greater regional area.

The focus group agenda, informed by the quantitative data, will be presented by two researchers as the initial discussion point. Participants in the patient group will be asked to discuss their own experience of accessing ED with a non-urgent condition and to reflect on the earlier findings. Subsequent to this, the second focus group, comprised of GPs and primary care clinicians will be presented with the analysed quantitative trends and with themes identified during the analysis of the patient focus group. Discussion will seek to understand GP experiences in referring patients with non-urgent conditions to the ED and the health requirements of this group.

Both focus groups will be audio recorded and transcribed. These transcriptions will then be analysed using an inductive approach in order to identify emerging themes.

### Objective 3: Translation of research knowledge into health service recommendations

The third objective for this project will aim to translate knowledge gained from the previous quantitative and qualitative stages. This will be done through presentation of the findings at a local forum involving primary and acute care clinicians, academics, patient representatives and policy makers. The goal will be to share the knowledge obtained during the first two objectives and to engage key stakeholders in the process of translating this into health service recommendations, policy and planning. The notion of knowledge translation has arisen out of concern for the time taken for research to influence healthcare. It is hoped that through engagement with local ED clinicians (nursing and medical), general practitioners, practice nurses, hospital administrators, patient representatives, academics, hospital administrators, policy makers and government officials, the process of research translation will facilitate clear identification of service needs and future planning of a suitable, sustainable needs-based and patient-focused health service model. The purpose of this stage will be to discuss project findings and identify a service model designed to appropriately meet community needs and to facilitate timely access to services; the right service, at the right time and in the right place.

## Discussion

The findings of this project will add to a body of research being conducted in Tasmania to address the issue of ED crowding. Previous research has demonstrated that a significant proportion [24] of patients with non-urgent conditions had attempted to access alternative services before arriving at the ED, with many stating they would prefer to be managed by their GP, and over a quarter of this patient group being referred (formally or informally) by their GP, therefore indicating the ED is not the preferred option for many patients. In this regional Australian city, if the 31% of non-urgent ED presentations could have been assessed and managed at an alternative service, up to 8000 presentations per year could have been directed away from the ED.

The research team anticipate the findings from this project will clearly identify local issues faced by patients who have attempted to seek medical attention from their GP, yet, are directed to the ED where they are triaged as non-urgent. These findings will be relevant within the local context and will be used to inform future service models aimed to provide the right service at the right time in the right place, thus improving equitable access to healthcare.

## Abbreviations

ATS: Australasian triage scale
ED: Emergency department
GP: General practitioner
ITS: Interrupted time series
THS: Tasmanian Health Service
